# Estimating the causal effect of dexamethasone versus hydrocortisone on the neutrophil- lymphocyte ratio in critically ill COVID-19 patients from Tygerberg Hospital ICU using TMLE method

**DOI:** 10.1186/s12879-024-10112-w

**Published:** 2024-11-29

**Authors:** Ivan Nicholas Nkuhairwe, Tonya Marianne Esterhuizen, Lovemore Nyasha Sigwadhi, Jacques Lukenze Tamuzi, Rhoderick Machekano, Peter S. Nyasulu

**Affiliations:** 1 Division of Epidemiology and Biostatistics, Department of Global Health, Stellenbosch University, 3rd Floor, Education Building, Francie Van Zijl Drive, Parow, Cape Town, 7500 South Africa; 2 Division of Epidemiology and Biostatistics, School of Public Health, Faculty of Health Sciences, University of the Witwatersrand, Johannesburg, South Africa; 3 Biostatistics Research Group (BRG), Mikro Park, Kuilsriver, Cape Town, South Africa

**Keywords:** Targeted maximum likelihood estimation, Neutrophil-lymphocyte ratio, COVID-19, Corticosteroids, Dexamethasone, Hydrocortisone, Targeted learning

## Abstract

**Background:**

Causal inference from observational studies is an area of interest to researchers, advancing rapidly over the years and with it, the methods for causal effect estimation. Among them, Targeted Maximum Likelihood estimation (TMLE) possesses arguably the most outstanding statistical properties, and with no outright treatment for COVID-19, there was an opportunity to estimate the causal effect of dexamethasone versus hydrocortisone upon the neutrophil-lymphocyte ratio (NLR), a vital indicator for disease progression among critically ill COVID-19 patients.

**Methods:**

TMLE variations were used in the analysis. Super Learner (SL), Bayesian Additive Regression Trees (BART) and parametric regression (PAR) were implemented to estimate the average treatment effect (ATE).

**Results:**

The study had 168 participants, 128 on dexamethasone and 40 on hydrocortisone. The mean causal difference in NLR on day 5; ATE [95% CI]: from SL-TMLE was − 0.309 [-3.800, 3.182] BART-TMLE 0.246 [-3.399, 3.891] and PAR-TMLE 1.245 [-1.882, 4372]. The ATE of dexamethasone versus hydrocortisone on NLR was not statistically significant since the confidence interval included zero.

**Conclusion:**

The effect of dexamethasone is not significantly different from that of hydrocortisone on NLR in critically ill COVID-19 patients admitted to ICU. This implies that the difference in effect on NLR between the two drugs is due to random chance. TMLE remains an outstanding approach for causal analysis of observational studies with the ability to be augmented with multiple prediction approaches.

**Supplementary Information:**

The online version contains supplementary material available at 10.1186/s12879-024-10112-w.

## Introduction

Causal inference can be defined as attaching a causal relationship between factors. Whilst the definition is not so informative, one attains the concepts of causal inference throughout their early learning experiences such as, a toddler learning not to touch a cooking pot. It is through these self-developed causal concepts that one designs control of their interactions with the factors around them [1, 2].

Causal inference from observational studies is advancing in epidemiology and with it, causal effect estimation methods. Targeted Maximum Likelihood Estimation (TMLE) is one of the most recent approaches and arguably the best based off its superior statistical properties [3].

TMLE is doubly robust and returns efficient, unbiased estimates for causal effect estimation. It can incorporate numerous algorithms including machine learning algorithms and is robust to outliers and sparsity. These outstanding statistical properties make it the estimator of choice given the complex nature of observational studies [3].

The major handicap of observational studies pertaining to causal inference is that unlike randomised controlled trials, treatment assignment is not randomised which gives rise to confounding bias. A solution to this impasse is then assuming that treatment was assigned at random conditional on measured covariates. Hence, this study used a COVID-19 observational study relying upon the assumption that it approximated to a conditionally randomised experiment [2].

### Motivating COVID-19 application

Coronavirus disease 2019 (COVID-19) caused by the novel SARS-Cov-2 (severe acute respiratory syndrome coronavirus 2) that originated from China, wreaked havoc across the world leaving in its wake unprecedented massive loss of human lives [4, 5].

SARS-CoV-2 on infiltrating its host, causes in its extreme form, alveolar damage with microvascular thrombosis. COVID-19 progression is characterised by a complex immune response leading to hyperinflammation, also known as a cytokine storm. This hyperinflammation further incapacitates the immune response leading to severe disease or even death [5, 6].

During the COVID-19 pandemic, various therapies were administered to arrest the escalation to mortality and among them, was corticosteroid therapy, chloroquine, hydroxychloroquine, lopinavir, etc. With no outright COVID-19 medication and the efficacy of each of these therapies under debate, corticosteroid therapy was associated with a beneficial impact on mortality risk of critically ill patients with the hyperinflammatory COVID-19 phenotype [7–9].

Corticosteroids are anti-inflammatory medicine potentially explaining the beneficial impact on mortality risk [10]. However, the specific impact of corticosteroids on the Neutrophil-Lymphocyte ratio (NLR) is of importance, since NLR is not only a good predictor for assessment of disease severity and mortality in patients with COVID-19, but also associated with the laboratory indicators related to disease conditions [11].

NLR at admission is a good quantitative clinical measure to discern between high and low mortality risk as well as a better response to corticosteroid therapy. It has been shown that corticosteroid therapy in patients admitted with NLR values above 6.11 corresponding to higher mortality risk is associated with reduced mortality while for patients admitted with NLR values less than or equal to 6.11, corticosteroid therapy did not reduce mortality risk [12]. Dexamethasone or hydrocortisone have different beneficial effects on the mortality risk in critically ill COVID-19 patients [12]. Therefore, it is crucial to determine their causal impact on the NLR, a predictor for mortality in critically ill COVID-19 patients [11–14].What better way to do this than to harness the exceptional statistical properties of TMLE to estimate the causal effect of these corticosteroids? This study was interested in the application of TMLE variations to draw causal inference from an observational study.

## Methods

### Study design

This was a retrospective observational cohort study conducted at Tygerberg Hospital (TBH) during the first two waves of the COVID-19 pandemic between 27 March 2020 and 10 February 2021. The TBH is a 1380-bed hospital that serves as the main teaching hospital for Stellenbosch University Faculty of Medicine and Health Sciences. TBH was designated as a centre for COVID-19 management with additional critical care services. It provides tertiary services to around 3.5 million people.

### Study population and sample size

The main study had 490 participants, and our study included data from 168 adult patients admitted with severe COVID-19 pneumonia. The study included participants were on either hydrocortisone or dexamethasone, no switching of medication and had complete profiles between day 1 and 5. The diagnosis was confirmed with a positive SARS-CoV-2 polymerase chain reaction (PCR). Details regarding admission criteria to ICU are documented in the Western Cape Government’s provincial guidelines [15].

Covariates considered were age at admission, gender, ventilation status, co-morbidities such as asthma, hypertension, chronic kidney disease, hyperlipidaemia, and HIV status. Smoking status and C-reactive proteins were recorded but had a lot of missingness and were dropped. Comorbidities were considered since they affect NLR hence, potential confounders. The main outcome or response variable was NLR at day 5.

### Data collection

Clinical data was extracted from ICU clinical notes and entered into a REDCap^®^ (Research Electronic Data Capture, Stellenbosch, South Africa) database, a secure web application. Laboratory data were imported from the National Health Laboratory Service (NHLS) Laboratory Information System (TrakCare^®^ Lab Enterprise) onto the REDCap database. Data quality assurance was undertaken by the research assistants and later verified by the supervisor of the research team to ensure data quality before analysis. Detailed information about the clinical parameters is defined in the previously published articles [16, 17].

### Targeted maximum likelihood estimation

TMLE incorporates a two-stage approach. The first step involves estimating an initial outcome model function then finally, a targeting step which updates the initial estimate to return an unbiased and efficient estimator of the target parameter [18].

TMLE combines the strengths of both G-computation and propensity score-based methods. On one hand, G-computation estimates an outcome model function which then is used to generate potential outcomes (counterfactual outcomes) under the different treatment scenarios for each individual. The difference between the average of these counterfactual outcomes is the causal effect. Propensity score-based methods on the other hand apply weighting i.e. estimate the probability of treatment assignment given observed covariates for each individual and these weights are further applied when estimating the causal effect [19].

TMLE starts with a G computation-like step: estimate an outcome model function and use it to predict potential outcomes. The targeting step subsequently estimates the exposure mechanism (treatment assignment given measured covariates), which is used to update the initial outcome model function using a propensity score-based clever covariate H. The covariate H is judiciously chosen to enhance the accuracy of the estimation process, hence its description as clever. The second step concludes with causal effect estimation using the updated estimates [19, 20].

### Super learner (SL) and TMLE

The initial step involved creating a node list where variable roles were defined into W (covariates), A (treatment) and Y (outcome) [21].

The next step involved defining learners which took the form of a list of Super Machine Learning with Pipelines (R/sl3)^22^ learners. Instead of selecting a learner for each likelihood factor to be estimated i.e. The initial outcome model function and propensity score function, as illustrated by Van der Laan et al., 2022 [21], a stack of suitable base learners taking into consideration the data type of the outcome (continuous) and treatment (binary) was defined based off criteria from Phillips et al.,2022 [23]. Two meta-learners (Nonnegative linear least squares, Nonlinear Optimization via Augmented Lagrange (NoDSL)) were used in one estimation and then another estimation used the discrete meta-learners (DSL). This was done to get an appreciation of the difference in the meta-learners, with the discrete ones applying a winner take it all approach while the former take the average of the base learners. This was done due to limited computational resources to enable the defining of ensemble learners within the base learners where the discrete meta-learners would be used to evaluate the defined learner.

### BART and TMLE

It involved defining a list of confounders, treatment variable, outcome variable, method (TMLE) to fit the outcome model function, method (BART) to fit the treatment assignment mechanism, a common support rule (chi-square) to exclude any observations based on the ratio of the variance of posterior predicted counterfactuals to the posterior variance of the observed condition. This ratio follows a Chi-Squared distribution with one degree of freedom under the null hypothesis of equal distributions and the estimated ATE.

The method to fit the outcome model function; TMLE fit the outcome model function using BART and further adjusted with TMLE. For the case of the treatment assignment mechanism, it was fit using BART [24]. Elaborate explanations on the background processes of the package can be found at the *webpage*^1^.

### Parametric regression and TMLE (PAR-TMLE)

Initial step involved transforming the continuous outcome to be bounded between 0 and 1.

Logistic regression models were then implemented to obtain the outcome model function and potential outcomes. This was followed up by prediction of propensity scores by fitting a logistic regression model to the binary treatment.

Clever covariates corresponded to treatment assignment 1 (dexamethasone) and 0 (hydrocortisone) were then estimated and the fluctuation parameter ϵ estimated. (See the Appendix I: Simplified mathematical illustration of TMLE for explanation of the clever covariates).

These were then used to update the initial predictions of potential outcomes, and a mean difference calculated and rescaled to give the average treatment effect. Then 95% confidence intervals were estimated.

**Fig. 1:**
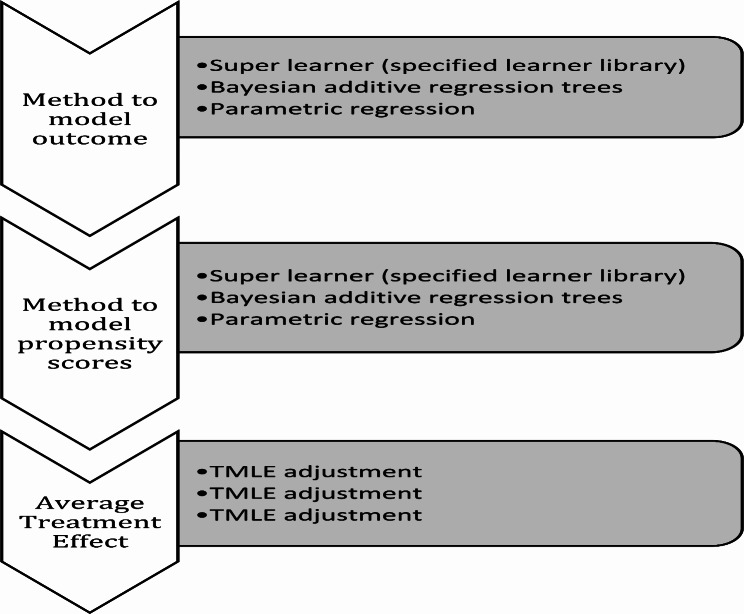
Flow diagram illustrating the implementation of TMLE variations

The manual implementation of TMLE followed the illustrations from Luque-Fernandez et al., 2018 and Karim & Frank, 2021 [25, 26]. Figure 1 illustrated the implementation of TMLE variations.

### Statistical analysis

Descriptive statistics, such as frequency, percentage, and median with interquartile range (IQR), were used to summarize the patient characteristics. Chi-square test, Fisher’s exact test and Wilcoxon rank sum test were used to compare the patient characteristics between the dexamethasone and hydrocortisone groups. Statistical significance level for these tests was 5%.

Super learner, BART, and parametric regression methods were used to obtain the initial outcome model function estimate. Then, the respective estimates used to implement TMLE.

SL and TMLE were implemented cohesively under the tmle3 [27] and sl3 [22] libraries in R software. The node list, tmle3_specification object and defined learner list were passed to the tmle3 function that returned the average treatment effect estimate.

BART and TMLE were implemented using the R package bartCause [24]. The defined arguments were passed to the bartCause::bartC function, which returned the average treatment effect estimate.

Missing data was imputed using multiple imputation with chained equations in the R MICE package. Continuous variables were imputed using predictive mean matching, binary variables using logistic regression and categorical variables with more than two unordered levels using polytomous regression. Passive imputation was done for missingness in the response variable NLR on day 5 and NLR on day 1 to remove circularity [28].

A sensitivity analysis where the original dataset is used as is with its missing values was not possible since the TMLE adjustment does not accept missingness. When the data is processed for TMLE in the tmle3 R package, continuous variables are imputed with median and discrete variables, mode. Any observation with a missing value for the treatment variable is dropped and missing outcomes are handled by automatic calculation of inverse probability of censoring weights (IPCW-TMLE) [21]. In the case of BART-TMLE, missingness is also not compatible with method tmle [24]. However, a complete case analysis was done where any observation with missingness was dropped, and only complete observations were considered.

Appendix II: Summary statistics table for all data sets shows the characteristics of original data (OBS), imputed data (IMP) and complete case data (CC).

All analysis was done in R software version 4.2.0 and RStudio build 353 [29, 30].

## Results

Table 1 shows the cross tabulation and summary statistics of the one hundred and sixty-eight (168) participants, 56% (94/168) were females, and 44% (74/168) were males. 76% (128/168) received dexamethasone, and 24% (40/168) were on hydrocortisone.

The median age of the participants was 57.0 (IQR: 48.0–63.0) years. The median NLR at baseline was 12.6 (IQR: 8.0-18.1) and the median NLR on day 5 was 15.9 (IQR: 11.0-23.6). There was an increase in NLR from baseline to day 5 with the median NLR at baseline being 12.6 (IQR: 8.0-18.1) and the median NLR on day 5 being 15.9 (IQR: 11.0-23.6). This increase was evident in both treatment groups. The median NLR at baseline was 9.1 (IQR: 7.3–15.5) and that on day 5 was14.4 (IQR: 11.1–20.4) in the hydrocortisone group while NLR at baseline was 13.1 (IQR: 9.5–18.6) and 17.3 (IQR: 11.0-24.9) in the dexamethasone group.

The categorical variables were not associated with the treatment, with p-values greater than 0.05 from their respective Pearson’s chi-squared tests and Fisher’s exact tests.

There was no statistically significant association between corticosteroid type and, ventilation status (*p* = 0.311), gender (*p* = 0.889), HIV status (*p* = 0.5105), hyperlipidaemia status (*p* = 0.4115), chronic kidney disease status (*p* = 0.5113), asthma status (*p* = 1.00), diabetes mellitus status (*p* = 0.277), hypertension status (*p* = 0.184).

Age at admission (*p* = 0.199) and NLR on day 5 (*p* = 0.349) were equally distributed between dexamethasone and hydrocortisone groups with p values greater than 0.05 from their respective Wilcoxon rank sum tests. However, median NLR at baseline between the dexamethasone and hydrocortisone groups was statistically different (13.1 versus 9.1 respectively, *p* = 0.008).

**Table 1:** Cross-tabulation of other variables with corticosteroid type

Variable	Variable label	Corticosteroids type		Total	*P* value (test)
		Hydrocortisone	Dexamethasone		
Ventilation status	Non-invasive	35 (87.5%)	103 (80.47%)	138 (82.14%)	p value: 0.3108 (Pearson’s Chi-squared test)
	Invasive	5 (12.5%)	25 (19.53%)	30 (17.86%)	
Age at admission	Median [IQR]	57.0 [44.8;62.0]	56.5 [49.0;63.0]	57.0 [48.0;63.0]	p value: 0.1998 (Wilcoxon rank sum test)
Gender	Female	22 (55%)	72 (56.25%)	94 (55.95%)	p value: 0.8894 (Pearson’s Chi-squared test)
	Male	18 (45%)	56 (43.75%)	74 (44.05%)	
Hypertension status	No	18 (45%)	41 (32.03%)	59 (35.12%)	p value: 0.1835 (Fisher’s Exact Test for Count Data)
	Yes	22 (55%)	87 (67.97%)	109 (64.88%)	
	Unknown	0 (0%)	0 (0%)	0 (0%)	
Hyperlipidaemia status	No	37 (92.5%)	111 (86.72%)	148 (88.1%)	p value: 0.4115 (Fisher’s Exact Test for Count Data)
	Yes	3 (7.5%)	17 (13.28%)	20 (11.9%)	
	Unknown	0 (0%)	0 (0%)	0 (0%)	
Diabetes Mellitus	No	14 (35%)	58 (45.31%)	72 (42.86%)	p value: 0.2766 (Fisher’s Exact Test for Count Data)
	Yes	26 (65%)	70 (54.69%)	96 (57.14%)	
	Unknown	0 (0%)	0 (0%)	0 (0%)	
HIV status	No	32 (80%)	101 (79.68%)	133 (79.17%)	p value: 0.5105 (Fisher’s Exact Test for Count Data)
	Yes	6 (15%)	14 (10.16%)	20 (11.90%)	
	Unknown	2 (5%)	13 (10.16%)	15 (8.93%)	
Chronic Kidney disease	No	36 (90%)	119 (92.97%)	155 (92.26%)	p value: 0.5113 (Fisher’s Exact Test for Count Data)
	Yes	4 (10%)	9 (7.03%)	13 (7.74%)	
	Unknown	0 (0%)	0 (0%)	0 (0%)	
Asthma	No	39 (97.5%)	122 (95.3.1%)	161 (95.83%)	p value: 1.0000 (Fisher’s Exact Test for Count Data)
	Yes	1 (2.5%)	6 (4.69%)	7 (4.17%)	
	Unknown	0 (0%)	0 (0%)	0 (0%)	
Neutrophil-Lymphocyte Ratio at baseline	Med [IQR]	9.1 [7.3;15.1]	13.1 [9.5;18.6]	12.6 [8.0;18.1]	p value: 0.0081 (Wilcoxon rank sum test)
Neutrophil-Lymphocyte Ratio on day 5	Med [IQR]	14.4 [11.1;20.4]	17.3 [11.0;24.9]	15.9 [11.0;23.6]	p value: 0.3499 (Wilcoxon rank sum test)

Table 2 shows estimates from the three TMLE approaches with their respective 95% confidence intervals or 95% credible interval per BART-TMLE fit. They are shown per dataset and meta-learner used in the case of SL-TMLE. The MICE imputed dataset was considered as the main result (discrete meta-learner for SL-TMLE).

The average treatment effect (ATE) which is equivalent to the causal difference in mean NLR on day 5 if all participants were on dexamethasone versus hydrocortisone is -0.309 [-3.800, 3.182] when applying SL-TMLE (Table 2).

The causal difference in mean NLR on day 5 if all participants were on dexamethasone versus hydrocortisone when applying BART-TMLE is 0.246 [-3.399, 3.891] (Table 2).

PAR-TMLE returns a causal difference in mean NLR on day 5 if all participants were on dexamethasone versus hydrocortisone of 1.245 [-1.882,4372] (Table 2).

The causal difference in mean NLR on day 5 from all three methods if all participants were on dexamethasone versus hydrocortisone is statistically insignificant with intervals containing zero.

**Fig. 2:**
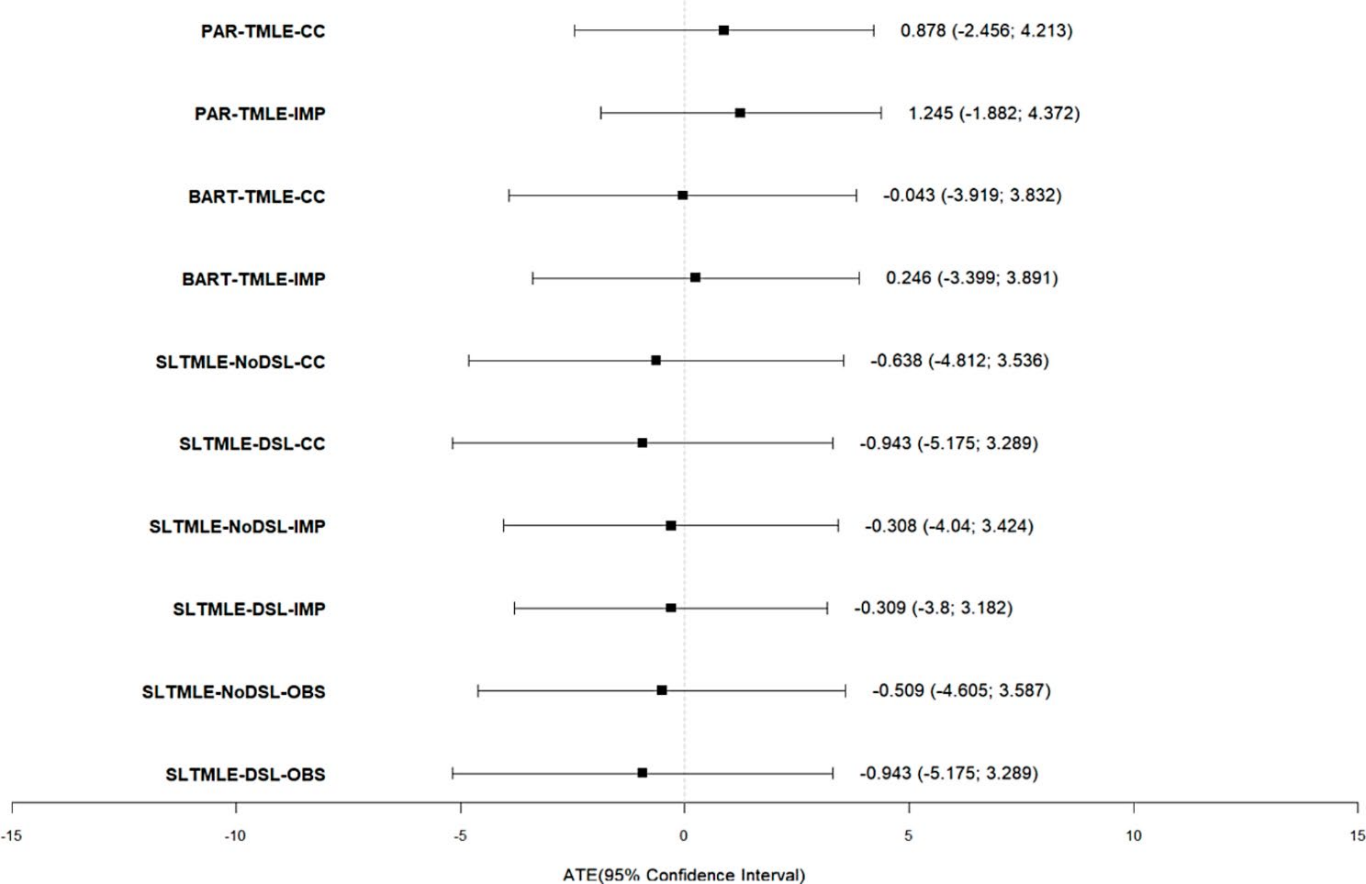
Forest plot of ATE estimates from TMLE variations specifying the datasets and meta-learners used

Figure 2 shows that the intervals from all three methods overlap, meaning that there is no statistically significant difference between the ATE estimates from the three different methods.

**Table 2:** ATE estimates with their 95% confidence intervals in brackets (*se* standard error)

Method	Observed data as is		MICE imputed data		Complete case data	
SUPERLEARNER-TMLE	DSL	No-DSL	DSL	No-DSL	DSL	No-DSL
	-0.943 *2.159* ^*s.e*^ [-5.175,3.289]	-0.509 *2.090* ^*s.e*^ [-4.605,3.587]	-0.309 *1.781* ^*s.e*^ [-3.800,3.182]	-0.308 *1.904* ^*s.e*^ [-4.040,3.424]	-0.943 *2.159* ^*s.e*^ [-5.175,3.289]	-0.638 *2.129* ^*s.e*^ [-4.812,3.536]
BART-TMLE	Not Applicable		0.246 *1.859* ^*s.e*^ [-3.399,3.891]		-0.043 *1.977* ^*s.e*^ [-3.919,3.832]	
PARAMETRIC-TMLE	Not Applicable		1.245 *1.595* ^*s.e*^ [-1.882,4.372]		0.878 *1.701* ^*s.e*^ [-2.456,4.213]	

Diagnostics run on BART-TMLE as seen in Appendix III: Imputation diagnostics and BART common support diagnostics show that the model fit suitably.

## Discussion

This study’s objective was to estimate the causal effect of dexamethasone versus hydrocortisone on the neutrophil-lymphocyte ratio in critically ill COVID-19 patients from Tygerberg Hospital ICU, using TMLE method. The study implemented three variations of TMLE, i.e., SL-TMLE, BART-TMLE and PAR-TMLE in estimating the ATE.

The ATE estimates from the three variations of TMLE were statistically insignificant hence, implying that the effect of dexamethasone is not significantly different from the effect of hydrocortisone on the Neutrophil-Lymphocyte ratio in critically ill COVID-19 patients admitted to ICU.In light of the negligible estimates of the ATE, there is insufficient evidence to reject the null hypothesis of a zero average treatment effect. Other than the known better potency and longer lasting action of dexamethasone compared to hydrocortisone [31], there is no literature comparing the corticosteroids’ effects on NLR. This further highlights the need for research in this area.

Additionally, the increase in NLR on day 5 from baseline implies that there is a spike in NLR post administration of corticosteroids. This is due to well documented corticosteroid-induced lymphopenia and neutrophilia [32]. This reduction in lymphocytes and increase in neutrophils results into the high NLR on day 5 post administration of corticosteroids.

The evaluation for which method was more efficient was beyond the scope of this study since it requires a simulation study to fully assess each method. However, results from a data analysis competition by Dorie et al., 2019 [33] found BART-TMLE to outperform SL-TMLE in terms of coverage and average interval length. However, the same study found augmenting most causal estimation methods with TMLE adjustment improved performance which further underlines the TMLE properties of flexibility and ability to return efficient, unbiased estimates.

In this study, parametric regression with TMLE returned a negligibly narrower 95% confidence interval which could misleadingly imply better precision than SL-TMLE and BART-TMLE that had relatively similar confidence intervals. This contradicts the findings from the study that found methods that flexibly model the outcome model function outperform those that do not [33]. However, the confidence intervals overlapped, hence implying no statistical difference between the ATE estimates from all three variations.

The negligible difference in confidence intervals of PAR-TMLE is misleading and a possible explanation for this anomaly could lie in the data generating process. The data used for analysis in this study had missingness in the covariates however, not in the treatment variable and less than 10% in the outcome of interest. Data was imputed under the MICE package where the model for imputing binary variables and categorical variables with more than two unordered levels was logistic regression and polytomous regression, respectively. The continuous variables were imputed using predictive mean matching. As such, implementing logistic regression in the estimation of the outcome model function could be closer to the data generating distribution hence presenting better predictions than the discrete Super Learner that selects from a stack of algorithms and then TMLE adjustment is applied. In his *blog post*^2^, Van der Laan explains this precisely as, ”one first assumes a parametric likelihood to carry out imputations, and then one assumes a nonparametric model for the resulting full-data set and applies TMLE” which defeats the whole idea of targeted learning. It is also worth noting that imputation can lead to the underestimation of the true variance, resulting in narrower confidence intervals [34]. This is evident in the confidence intervals returned by PAR-TMLE-CC (where the complete case dataset is used) which are wider than those of PAR-TMLE-IMP.

Additionally, there is fluctuating ATE estimates and their respective 95% confidence intervals with the different meta-learners specified under the SL-TMLE framework. This is evidence of robustness since the varying 95% confidence intervals consistently overlap which implies that the estimates are not that statistically different.

The complexity of observational data, specifically regards the numerous covariates to be considered (high dimensionality) when modelling, inherently favours methods that are flexible [35]. i.e., methods that require less strict parametric assumptions and can simultaneously apply semi-parametric and parametric algorithms thereby detecting complex relationships easily. Van der Laan et al.,* 2022*, argues that over simplification with parametric assumptions introduces bias through model misspecification and offer the solution of super learning [21]. It is therefore, vital that extra attention be taken when selecting the method for estimating the outcome model function and propensity scores in causal analysis of observational data as more often than not, parametric regression won’t suffice and if possible, should be avoided.

The biggest challenge with super learner implementation lies with construction of the learner library and why one selects the learners they end up applying. Despite guidance from Phillips et al.,2022 [23], there is limited guidelines on what learners to consider, i.e., many learners are suggested for continuous outcomes or categorical outcomes, is it a personal preference on which learners make the list and how to fine tune them or otherwise? Therefore, this calls for a more elaborate guideline on application of these machine learning approaches.

## Conclusion

Targeted Maximum Likelihood Estimation is an outstanding and flexible approach for drawing causal inference from observational studies with the ability to be augmented with multiple prediction approaches. One of the properties highlighted by this study is its robustness, returning closely similar confidence intervals for the three ATE estimates.

The effect of dexamethasone is not significantly different from that of hydrocortisone on NLR in critically ill COVID-19 patients admitted to ICU. This implies that the difference in effect on NLR between the two drugs is due to random chance.

### Limitations

Due to computational constraints, the stack of learners implemented under SL-TMLE was restricted and could not include desired ensemble learners in the Discrete super learner approach.

The sample size could potentially have hindered the study from getting the best results. However, there is no information on what minimum sample size is when using TMLE with Super Learner or BART. Additional limitations were emphasized in the discussion section as outlined by Van der Laan et al.,* 2022* [21].

## Electronic supplementary material

Below is the link to the electronic supplementary material.

Supplementary Material 1

## Data Availability

The datasets generated and/or analyzed during the current study are not publicly available due to original data rights belonging to the main study but are available from the corresponding author on reasonable request pending approval from the main study principal investigator.

## Footnotes

1 https://search.r-project.org/CRAN/refmans/bartCause/html/bartc.html.

2 https://vanderlaan-lab.org/2019/05/10/imputation-and-missing-data-in-the-tmle-framework/.
