# Supplementary material for: Estimating the causal effect of dexamethasone versus hydrocortisone on the neutrophil- lymphocyte ratio in critically ill COVID-19 patients from Tygerberg Hospital ICU using TMLE method
Source: BMC Infect Dis. 2024 Nov 29;24:1365. doi: 10.1186/s12879-024-10112-w (PMC11606140; doi:10.1186/s12879-024-10112-w)

**Appendix I: Simplified mathematical illustration of TMLE**

Consider a binary treatment $A, A=\left\{ 1,0 \right\};$ 1 corresponding to treatment (exposed) and 0 control (unexposed). For this study, 1-dexamethasone, 0-hydrocortisone. Outcome $Y$ (either binary or continuous, where each individual $i , i=\{1,\ldots,n\}$ has a pair of potential outcomes, $Y_{i}^{a=0}$ for when unexposed and $Y_{i}^{a=1}$ for exposed, and measured covariates $W$ from an observational dataset; $O_{obs}=\left( W,A,Y \right)$ ^2,35^.

Causal inference from observational studies using TMLE is dependent on three assumptions. Consistency ( $Y_{i}^{a}=Y_{i}\forall i, with A=a$); the treatment status of an individual does not affect the potential outcome of others and treatment level is the same for all individuals at that level, known as Stable Unit Treatment Value Assumption (SUTVA), Conditional Exchangeability ($Y^{a}⫫A|W$ ) ; within levels or strata of W, the observed treatment and potential outcomes are independent and, Positivity ($Pr \left[ A=a | W=w \right]>0 \forall w, with Pr [W=w]\neq0$) ; within strata of W, each individual has a nonzero probability of receiving either exposure ^2,3^.

We can then define our parameter of interest, average treatment effect (ATE) as $\psi=E\left( Y^{a=1} \right)-E\left( Y^{a=0} \right)$ ,interpreted as the difference in mean outcomes between the treatment groups. However, under the untestable causal assumptions as is with observational studies, the ATE is calculated using the G-formula, $\psi=\mathbb{E}_{W}\left( \mathbb{E}\left( Y|A=1,W \right)\mathbb{-E}\left( Y|A=0,W \right) \right)$ , interpreted as the mean difference in outcome Y between the exposed and unexposed adjusted for measured covariates averaging over the distribution of measured covariates W^3,21,24,26,35^

Under TMLE, the initial step estimates the conditional expectation of outcome Y given the exposure and covariates alias outcome model function, $E\left[ Y|A,W \right]$ denoted as $\overline{Q}_{n}\left( A,W \right)$. Then, the targeting step involves estimation of the exposure mechanism (propensity scores), $g_{n}\left( A | W \right)\mathbb{=P}\left( A=1|W \right)$ which is then used to update the initial outcome model through a predefined formula.

The updating step uses the propensity score based H clever covariate, $H_{n}\left( A,W \right)=\frac{A}{g_{n}\left( A\mid W \right)}-\frac{A}{1-g_{n}\left( A\mid W \right)}$ in the predefined formula which also estimates epsilon ($\epsilon$), the fluctuation parameter which provides information on how much to change the initial estimate^19,21,35^.

The now updated estimate denoted as $\overline{Q}_{n}^{*}\left( A,W \right)$ can be used in the below equation to get the ATE

$\psi_{n}^{\mathcal{T}MLE}=\frac{1}{n}\sum_{i=1}^{n} \overline{Q}_{n}^{*}\left( 1,W_{i} \right)-\overline{Q}_{n}^{*}\left( 0,W_{i} \right)$ ^21,35^.

**Appendix II: Summary statistics table for all data sets**

| **Variable** | **Variable label** | **Original dataset (OBS)** | **Imputed dataset (IMP)** | **Complete Case (CC)** |
| --- | --- | --- | --- | --- |
| Corticosteroids type | Hydrocortisone | 40 (23.81%) | 40 (23.81%) | 38 (26.57%) |
|  | Dexamethasone | 128 (76.19%) | 128 (76.19%) | 105 (73.43%) |
| Ventilation status | Non-invasive | 136 (82.93%) | 138(82.14%) | 122 (85.31%) |
|  | Invasive | 28 (17.07%) | 30 (17.86%) | 21 (14.69%) |
|  | Missing values | 4 | 0 | 0 |
| Age at admission | Min / Max | 29.0 / 76.0 | 29.0 / 76.0 | 29.0 / 76.0 |
|  | Med [IQR] | 57.0 [49.0;63.0] | 57.0 [49.0;63.0] | 58.0 [50.0; 63.0] |
|  | Mean (std) | 55.2 (10.0) | 55.1 (10.0) | 55.7 (9.9) |
|  | Missing values | 6 | 0 | 0 |
| Gender | Female | 89 (54.94%) | 94 (55.95%) | 80 (55.94%) |
|  | Male | 73 (45.06%) | 74 (44.05%) | 63 (44.06%) |
|  | Missing values | 6 | 0 | 0 |
| Hypertension status | No | 58 (35.58%) | 59 (35.12%) | 49 (34.27%) |
|  | Yes | 105 (64.42%) | 109 (64.88%) | 94 (65.73%) |
|  | Unknown | 0 (0%) | 0 (0%) | 0 (0%) |
|  | Missing values | 5 | 0 | 0 |
| Hyperlipidaemia status | No | 144 (88.34%) | 148 (88.10%) | 125(87.41%) |
|  | Yes | 19 (11.66%) | 20 (11.9%) | 18 (12.59%) |
|  | Unknown | 0 (0%) | 0 (0%) | 0 (0%) |
|  | Missing values | 5 | 0 | 0 |
| Diabetes Mellitus | No | 72 (44.17%) | 72 (42.86%) | 63 (44.06%) |
|  | Yes | 91 (55.83%) | 96 (57.14%) | 80 (55.94%) |
|  | Unknown | 0 (0%) | 0 (0%) | 0 (0%) |
|  | Missing values | 5 | 0 | 0 |
| HIV status | No | 131 (80.37%) | 133 (79.17%) | 113 (79.02%) |
|  | Yes | 19 (11.66%) | 20 (11.9%) | 18(12.59%) |
|  | Unknown | 13 (7.98%) | 15 (8.93%) | 12 (8.39%) |
|  | Missing values | 5 | 0 | 0 |
| Chronic Kidney disease | No | 151 (92.64%) | 155 (92.26%) | 134 (93.71%) |
|  | Yes | 12 (7.36%) | 13 (7.74%) | 9 (6.29%) |
|  | Unknown | 0 (0%) | 0 (0%) | 0 (0%) |
|  | Missing values | 5 | 0 | 0 |
| Asthma | No | 157 (96.32%) | 161 (95.83%) | 138 (96.5%) |
|  | Yes | 6 (3.68%) | 7 (4.17%) | 5 (3.5%) |
|  | Unknown | 0 (0%) | 0 (0%) | 0 (0%) |
|  | Missing values | 5 | 0 | 0 |
| Neutrophil-Lymphocyte Ratio at baseline | Min / Max | 0.7 / 73.3 | 0.7 / 106.7 | 3.1 / 96.0 |
|  | Med [IQR] | 12.6 [8.0;18.2] | 12.6 [8.0;18.1] | 11.1 [8.2; 15.8] |
|  | Mean (std) | 15.2 (10.8) | 16.0 (13.8) | 21.6 (26.6) |
|  | Missing values | 13 | 0 | 0 |
| Neutrophil-Lymphocyte Ratio on day 5 | Min / Max | 1.7 / 59.5 | 1.7 / 78.8 | 6.8 / 95.2 |
|  | Med [IQR] | 15.6 [10.8;22.4] | 15.9 [11.0;23.6] | 14.1 [10.8; 19.9] |
|  | Mean (std) | 18.6 (11.0) | 19.4 (12.6) | 24.5 (26.2) |
|  | Missing values | 16 | 0 | 0 |

**Appendix III: Imputation diagnostics and BART common support diagnostics**

Figure 3; Convergence plots for NLR on day 5 and NLR at baseline showing that imputation was succesful (all points fall on the line of unity). Relationship between NLR and neutrophils, lymphocytes is preserved.


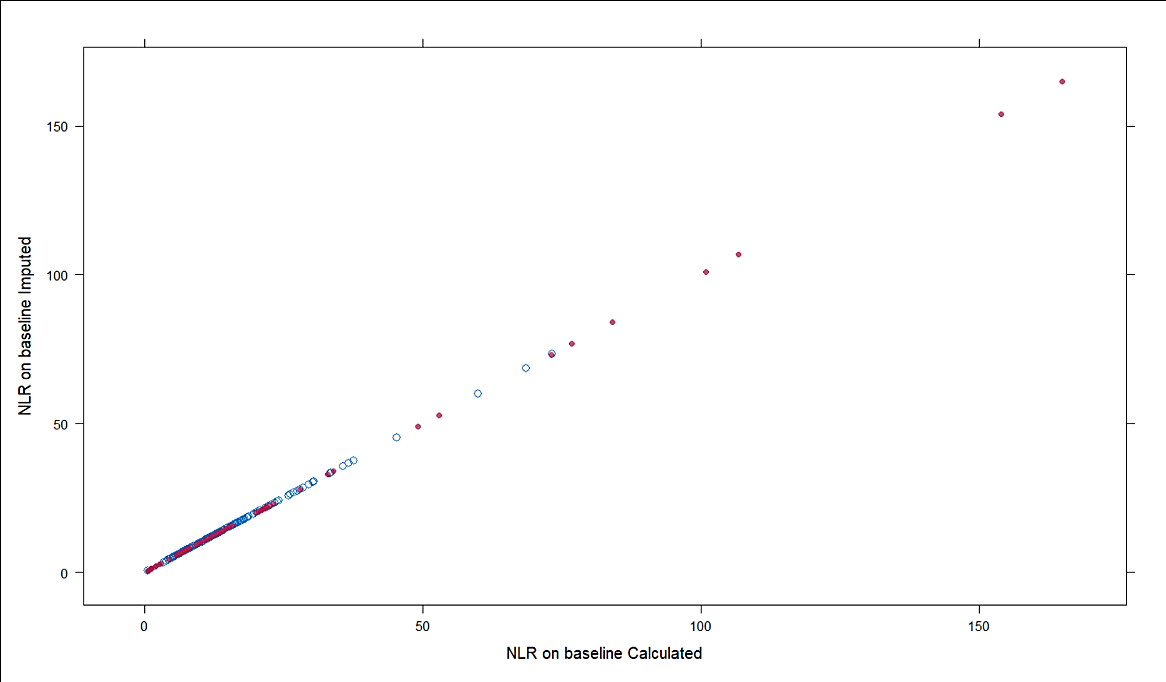

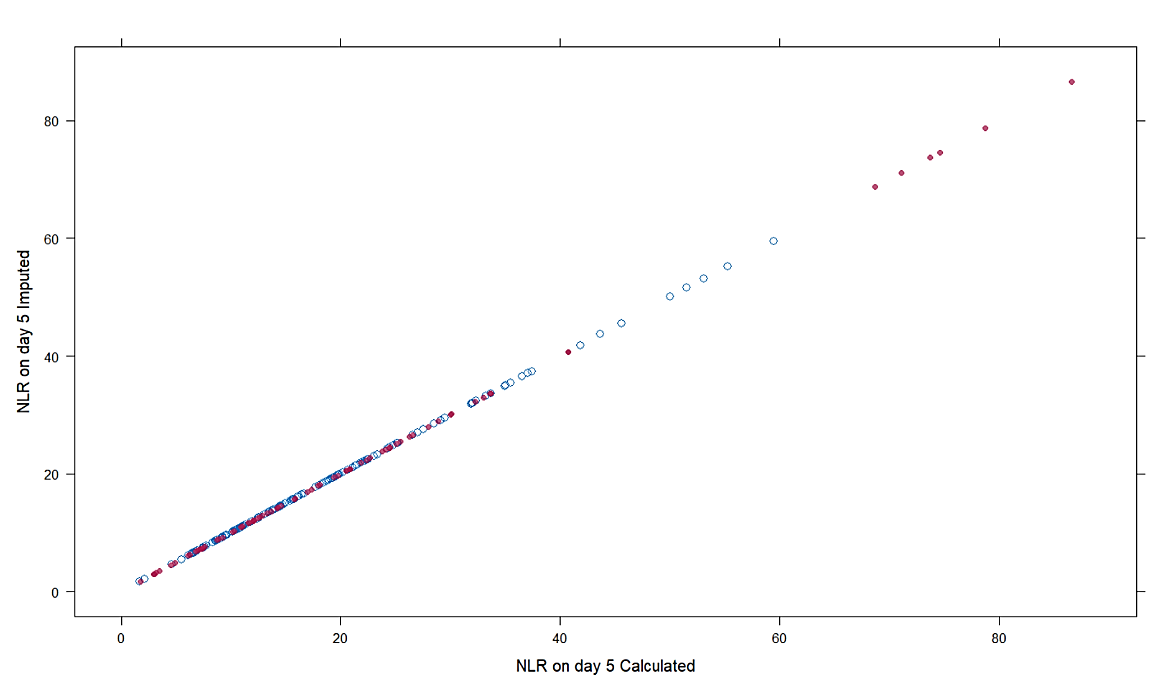


Figure 4; Trace lines: top is NLR on day 5, bottom is NLR at baseline.  Convergence can be inferred as the trace lines exhibit close alignment, indicating satisfactory stabilization.


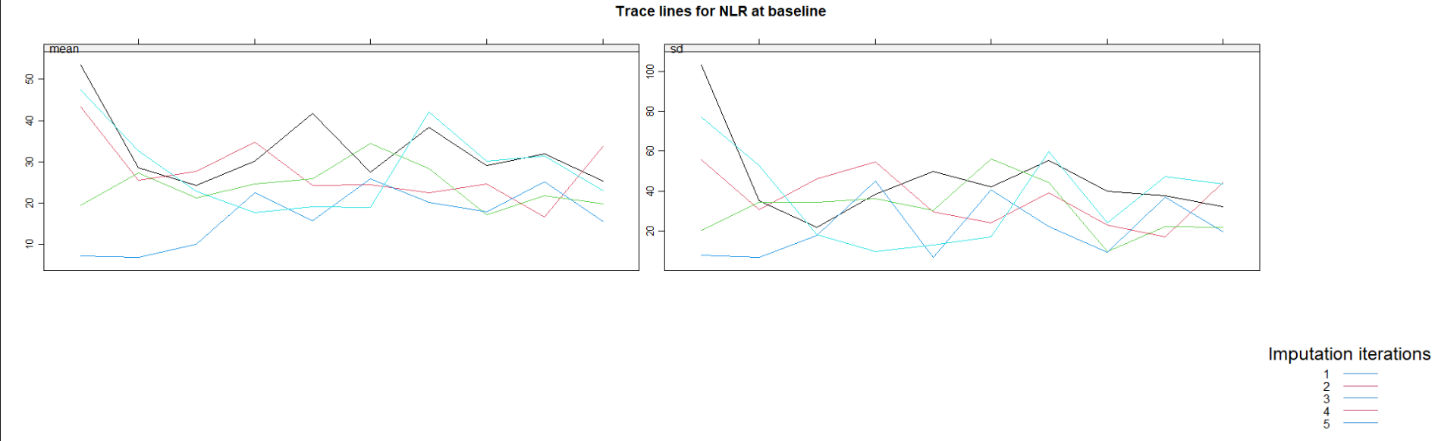

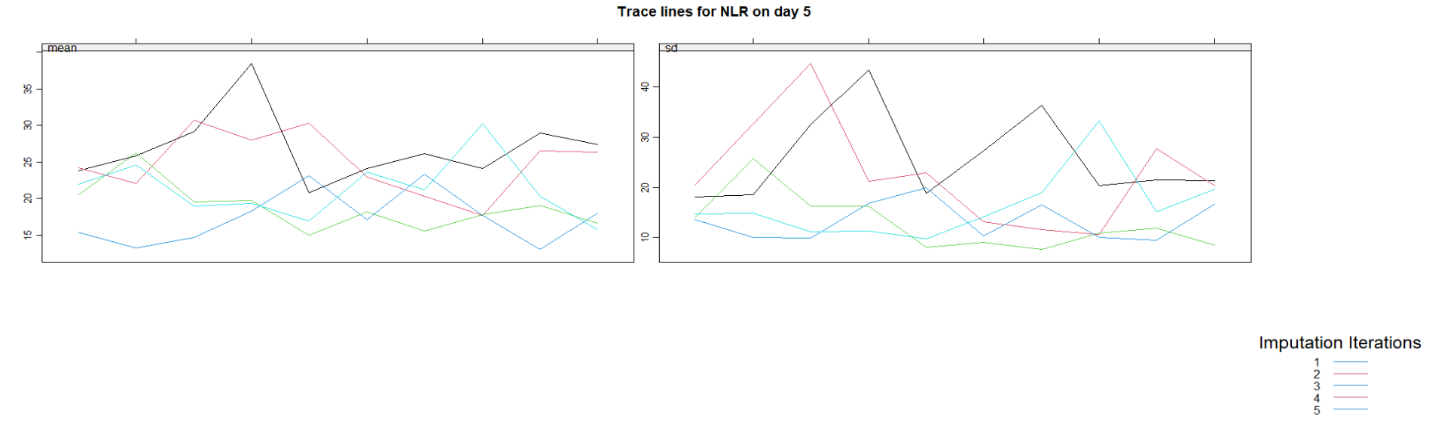


Figure 5; BART-TMLE common support checks showing both the IMP fit and CC fit (top and bottom respectively) fit well based on the chi-square check


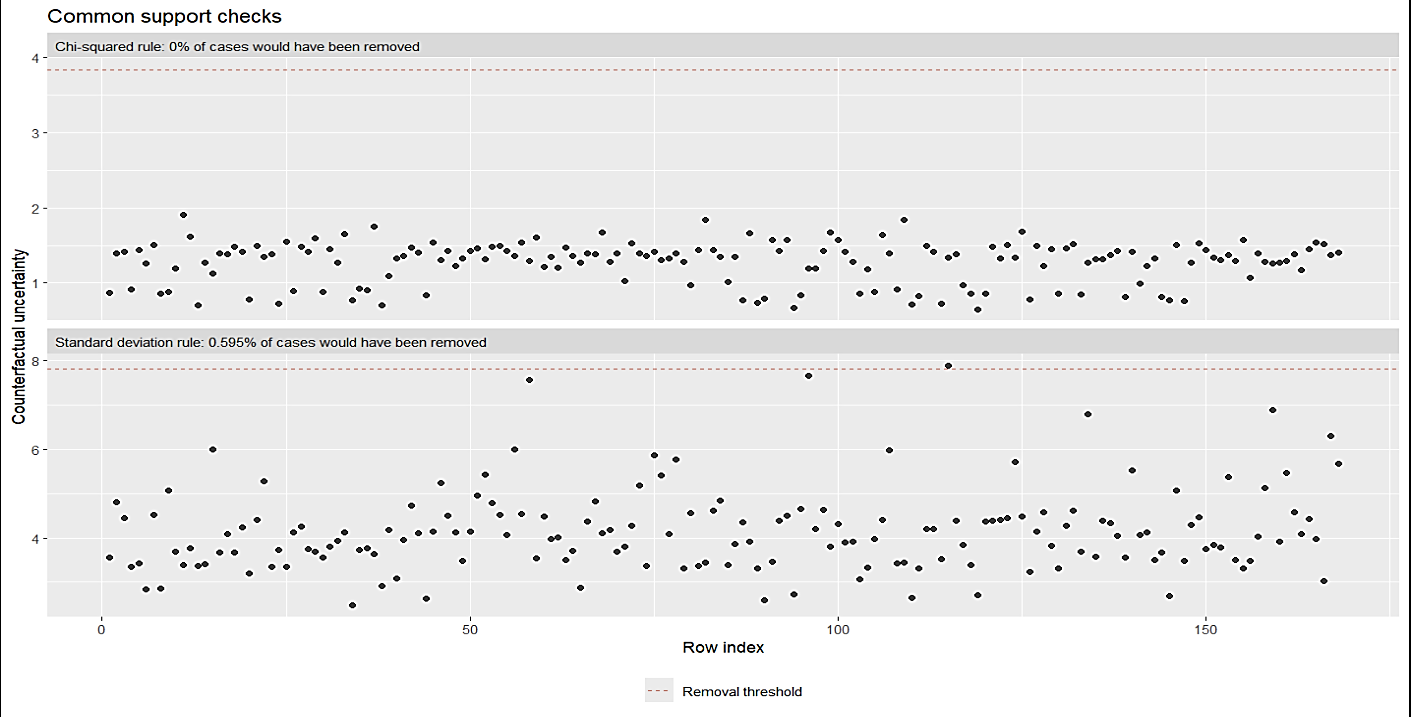

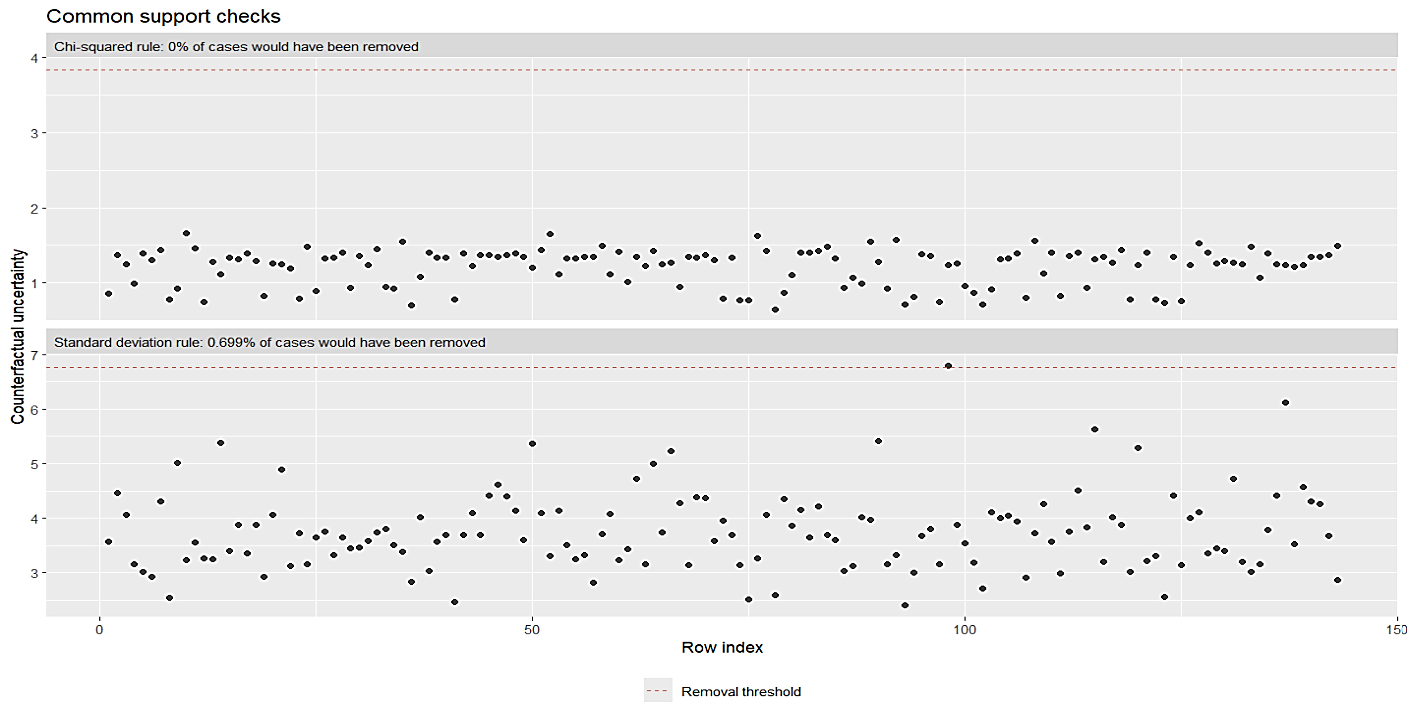

Supplement: Supplementary file 1 — Supplementary Material 1 [file 12879_2024_10112_MOESM1_ESM.docx]
